# The dark side of the ribosome life cycle

**DOI:** 10.1080/15476286.2022.2121421

**Published:** 2022-09-09

**Authors:** Sébastien Ferreira-Cerca

**Affiliations:** Regensburg Center for Biochemistry, Biochemistry III – Institute for Biochemistry, Genetics and Microbiology, University of Regensburg, Regensburg, Germany

**Keywords:** Ribosome, ribosome biogenesis, archaea, non-model organism, RNA

## Abstract

Thanks to genetics, biochemistry, and structural biology many features of the ribosome´s life cycles in models of bacteria, eukaryotes, and some organelles have been revealed to near-atomic details. Collectively, these studies have provided a very detailed understanding of what are now well-established prototypes for ribosome biogenesis and function as viewed from a ‘classical’ model organisms perspective. However, very important challenges remain ahead to explore the functional and structural diversity of both ribosome biogenesis and function across the biological diversity on earth. Particularly, the ‘third domain of life’, the archaea, and also many non-model bacterial and eukaryotic organisms have been comparatively neglected. Importantly, characterizing these additional biological systems will not only offer a yet untapped window to enlighten the evolution of ribosome biogenesis and function but will also help to unravel fundamental principles of molecular adaptation of these central cellular processes.

## 1. Lessons from ribosome biogenesis studies in model organisms

Ribosomes are universally conserved ribonucleoprotein complex carrying out the translation of mRNA into proteins. Despite a common general architecture and functional role within the cell, ribosome biogenesis and function show significant differences across the domains of life [1–3].

Among these differences is for example the set of conserved ribosomal constituents. The 33 universally conserved ribosomal (r-) proteins do not represent the full set of structural components forming the mature ribosomal subunits across the different domains of life [4]. In addition to these universally conserved core r-proteins, around 26 bacterial specific, or 34 archaeo-eukaryal specific r-proteins are known [2,4].

Another striking difference is that establishing a somewhat very similar functional entity requires much fewer ribosome biogenesis factors in bacteria than in eukaryotes. In the latter, a large expansion of the numbers of ribosome biogenesis factors facilitating ribosomal subunits maturation can be observed [2,5,6]. The molecular constraints or requirements that have emerged in eukaryotes to necessitate a remarkable complexification of the ribosomal subunit building process remain poorly understood to date.

Finally, translation initiation in model bacteria and eukaryotes is also divergent, as translation initiation in eukaryotes involved additional translation initiation factors [3].

Major differences are not only domain-specific, and some striking differences are also observed within the various domains of life. For example, the set of ribonucleases used for rRNA maturation in Gram-negative/-positive bacteria shows some variations. The RNase G/E family, which is important for some processing steps of the small ribosomal subunit rRNA, is absent in *B. subtilis* where, in this context, RNase J1 fulfils a similar function [7–9]. Similarly, there are notable differences in the eukaryotic rRNA maturation pathways as observed in yeast or human cells [10]. Moreover, the order of assembly/disassembly and/or the timing of action of some ribosome biogenesis factors, like the dimethyl transferase Dim1, occur at distinct steps of the yeast/human ribosome biogenesis pathways [11–13]. Even more striking is the molecular diversity and adaptation that can be observed in obligate parasites like mycobacterium or microsporidia. In these cases, some ribosome biogenesis factors seem to be absent (whether they have been lost or evolutionary selected against is so far unknown) and new compensatory mechanisms might have been implemented [9,14–16].

It is not fully surprising that our current view of ribosome biogenesis and function as studied in classical model organisms remains somewhat biased and does not allow us yet to fully appreciate the diversity of ribosome biology. Accordingly, revealing common and specific principles of ribosome biogenesis and function remains a challenging task for the field but will certainly help to better understand the molecular dance required for the formation and function of ribosomal subunits.

## 2. Crossing new (old) frontiers: ribosome biogenesis and function in archaea

### The discovery of archaea: impact on ribosome biology

At the end of the 1970s, Woese and colleagues, using comparative 16S rRNA cataloguing, described a new group of organisms, which is now known as the archaea, and proposed a third domain of life next to the bacteria and eukarya [17–21]. This seminal discovery and the basis of many subsequent phylogenetic analyses are therefore deeply connected to ribosome biology.

The archaeal discovery and the diversity of extremophile adaptations originally described in this domain of life have intrigued and attracted bold-minded scientists who wished to take advantage of their unusual biological properties [17,19]. For example, structural biologists have used archaeal proteins to determine molecular structures or reconstitute multi-subunit complexes that were albeit not easily accessible in other mesophilic organisms [22]. Accordingly, it is therefore not fully surprising that the first archaeal full genome sequencing of *M. jannaschii* was reported as early as 1996 [23], the same year of completion of the genome of one of the most commonly used eukaryotic model organisms, the yeast *S. cerevisiae* [24].

Archaea have contributed to our better understanding of the ribosome life cycle by helping to push forward the race to obtain the first high-resolution structure of ribosomal subunits. The pioneering work of James Lake´s laboratory, who structurally classified ribosomal subunits from various organisms by electron microscopy analysis, provided initial insights into common and specific structural features of ribosomal subunits across the domains of life [25,26]. Importantly, these studies were also challenging the three domains of life model proposed by Woese and colleagues. In fact, based on these initial structural analyses and additional information showing that some archaeal ribosomal subunits were more related to their eukaryotic counterparts, James Lake proposed the ‘Eocytes’ hypothesis [19,25–28]. This hypothesis suggests that the eukaryotic lineage has directly emerged from within the archaeal phylum, thereby proposing an alternative two domains division of the tree of life [19,25–28]. Likewise, pioneering work by Ada Yonath on *Haloarcula marismortui* ribosomal subunits, and the follow-up studies by other ribosome crystallography heroes, like Tom Steitz, belong probably among the most prominent examples of the contribution of archaeal research to our general understanding of ribosome biology [29–31]. For example, ribosomal subunits isolated from the halophilic archaeon *H. marismortui* have provided early critical insights into 50S ribosomal subunits structure and information on the binding of major antibiotics to this ribosomal subunit [32,33].

However, these early impactful contributions from the archaeal world on ribosomal biology research (and beyond) have been difficult to sustain. In contrast to *S. cerevisiae*, for which a systematic gene deletion project has been achieved shortly after the genome sequencing was completed [34], no such project has been performed in archaea. This was essentially due to the lack of robust and easy genetic manipulation tools available at the time but also because of the lack of a critical mass of scientists and resources necessary to carry out such an ambitious project. Still, some ribosome researchers were bold enough to continue to lay further the ground to genetically harness archaeal ribosomes or biochemically study the principle of archaeal translation and ribosome synthesis. Among these, the laboratory of Tom Steitz was pioneering rDNA deletion and the expression of mutant rDNA in Halophiles [32,35], while the laboratory of Paola Londei established *in vitro* reconstitution of translationally active archaeal ribosomal subunits from purified components [36–39] and additionally contributed with others, like the Dennis laboratory, to our initial understanding of ribosomal rRNA maturation in archaea [1,2,40–44]. Similarly, the early discovery of common rRNA modifications machineries, the s(no)RNPs, and the ability to reconstitute active archaeal s(no)RNPs complexes *in vitro* have been not only instrumental for our initial understanding of the biology of these complexes but remain important to reveal detailed mechanistic features of these conserved molecular machines [44–50].

Whereas the initial wave of deciphering the ribosome life cycle in archaea probably culminated with the atomic structure of the *H. marismortui* 50S ribosomal subunit [29,51], the scientific race to uncover the structural mysteries of the ribosome has probably required to focus on the most competitive model systems after all [52,53]. The difficulty to obtain high-resolution 3D structures of the 30S or 70S isolated from *H. marismortui* has probably been one of the major bottlenecks and led to a focus on other, more suitable, and ‘easier’ model organisms for further studies. Similarly, the key bottlenecks of limited genetic systems or easily accessible *in vitro* reconstitution systems have led to that only a small remaining core of archaeal aficionados kept analysing ribosome biogenesis and function in archaea, away from the last two decades of excitements seen around bacterial and eukaryotic ribosome biogenesis and function studies.

### ‘The archaea renaissance’

In recent years, a general increasing interest in studying archaeal biology has accelerated. Among the motors driving forward this archaeal ‘renaissance’, one can mention, on the one hand, the general interest to decipher the biology of CRISPR-Cas systems that are widely distributed in archaea [54,55], and on the other hand, probably one of the main contributors, the impact of metagenomics in sampling the microbiological diversity across the globe and the biological consequences of these discoveries [56,57]. Indeed, metagenomics-based genome reconstruction has unravelled new groups of archaea, among these, the Asgard archaea superphylum whose discovery has revitalized the discussion on the tree of life topology and the origin of eukaryotic cells from an archaeal ancestor [57–62]. As such it has revived the ‘Eocytes’ hypothesis and the two domains of life scenario originally put forward by James Lake [25–28], including the passionate discussion around these key topics [58,59,63].

In addition to these landmark studies that may have potentially stimulated scientists to address questions in this domain of life, it is also important to mention the steady development of more refined and robust genetic tools [64–67] facilitating functional studies, but also general methodological advances in this field. Probably one of the most remarkable and popular achievements is the rapid development of live-cell imaging of archaeal cells and fluorescent tagging withstanding the harsh growth conditions required for most genetically tractable archaea available so far [68–70]. Finally, one should not underestimate the impact and dedication of the vibrant and supportive community of archaeal biologists around the world determined to push boundaries.

Ribosome biogenesis and function in archaea are still poorly understood [1,2], but the possibility to answer key questions in this domain of life has not been as easy then as it is becoming now and will certainly be even easier in the coming years. The gap of knowledge between archaeal and the prototype models of bacterial and eukaryotic ribosome biogenesis and function remains to some extent immense, but there is no doubt that improved cultivation and genetic systems, functional analysis, combined with cryo-EM and *in cellula* cryo-electron tomography will help to close these gaps of knowledge and further reveal the common and specific principles of ribosome biology.

## 3. New world unleashed

The impact of metagenomics and culturomics probably remains still underestimated in many research areas [57,71,72]. However, it is very likely that these disciplines (will) have a key impact on our future global understanding of the biological diversity of the ribosome life cycles. Like the discovery of the Asgard archaea and other major archaeal groups [57,73,74], metagenomic analysis has also revealed an underappreciated biological diversity in bacteria. The candidate phylum radiation (CPR) represents a group of previously unknown organisms that may contribute up to 25% of the overall known bacterial diversity [73–75]. This sudden expansion of the bacterial and archaeal world offers exciting and invaluable resources for any ribosome biologist who wishes to explore the biological diversity and adaptation of this central process.

For now, genome mining remains the easiest and sometimes only way to access the mystery of many of these organisms and has already revealed interesting features, like the observed propensity of introns within the 16S and 23S rRNA sequences as well as the absence of various ribosomal proteins in CPR bacteria [75]. Access to these new organisms may remain limited and will highly depends on our ability to cultivate them in defined laboratory conditions and manipulate them genetically. Nevertheless, the combination of single cells -omics and the incoming *in situ* high-resolution revolution driven by cryo-ET, may enable us to crack open many of the little secrets of these yet-to-be-cultivated non-model organisms.

The discovery potential is enormous and can be easily illustrated by recent studies on ribosomal structures of parasitic ribosomal subunits that revealed how reductive evolution may shape ribosome biogenesis and function. Moreover, unleashing these ‘new worlds’ will also open a window towards better understanding of major evolutionary constraints of ribosome synthesis and function [9,14,15,60,73,76].

## 4. Outlook: the coming age of *in vivo* comparative ribosome biology

Comparative biology is probably as old as biology itself and remains a core discipline to understand phylogenetic relationships, the evolutionary history, and the biological diversity of key housekeeping processes.

The journey is a tedious but worthy one and our capacity to explore biological diversity is getting wider and easier every day; and yet many opportunities to follow new research avenues remain way too often unseized [77,78]. This is due, on the one hand, to scientific courage to follow new or alternative paths but, on the other hand, it is essentially impeded by multiple gatekeepers along the way, be it methodological, political, or due to scientific conformism. All these gatekeepers are sadly inhibiting the fundaments of innovation: curiosity and creativity, necessary to explore new frontiers.

In 2015, the same year where the Asgard archaea and CPR were first reported [61,75], Wiliam Sullivan wrote, independently of these discoveries, an essay entitled ‘The Institute for the Study of Non–Model Organisms and other fantasies’ [79]. This idea might be explored in very different ways [80] but should echo the need to build a sufficiently diverse scientific critical mass and raise public and politic awareness in order to hopefully leverage biological science to its full capacity.

## Data Availability

Data sharing is not applicable to this article as no new data were created or analyzed in this study.
